# Flexible and Navigable Suction Ureteral Access Sheath for Ureteral Stones: A Novel “Çapa” Surgical Technique

**DOI:** 10.5152/tud.2026.25073

**Published:** 2026-03-13

**Authors:** Rıfat Burak Ergül, M. Fırat Özervarlı, Vineet Gauhar, Steffi Kar- Kei Yuen, Olivier Traxer, Tzevat Tefik

**Affiliations:** 1 Department of Urology, Istanbul University Faculty of Medicine, Istanbul, Türkiye; 2 Department of Urology, Ng Teng Fong General Hospital, Singapore; 3 Department of Surgery, SH Ho Urology Centre, The Chinese University of Hong Kong, Hong Kong, China; 4 Department of Urology, Sorbonne University, Tenon Hospital, AP-HP, Paris, France

**Keywords:** Flexible and navigable suction, Minimally invasive urology, Ureteral stones, Ureteroscopy

## Abstract

**Objective:**

The flexible and navigable suction (FANS) ureteral access sheath (UAS) is an optimal innovation for retrograde intrarenal surgery (RIRS), proven to increase stone-free rates while reducing complications and the need for reintervention.^1^ This novel study aims to be the first to demonstrate the efficacy and reliability of FANS UAS in the treatment of ureteral stones.

**Material and Methods:**

A 61-year-old male patient presented with left flank pain. A non-contrast computed tomography (CT) scan revealed 4 stones of 9, 8, 6, and 6 mm in the distal ureter. A JJ stent was placed as part of initial management. One month later, the patient underwent ureteroscopy with FANS UAS to manage the ureteral stones.

**Results:**

The operation was successfully completed within 90 minutes. Ureteroscopy was performed using a 9.8 Fr single-use Redpine flexible ureteroscope, which was advanced through an 11/13 Fr, 40 cm FANS UAS. During laser lithotripsy, stone fragments were efficiently aspirated into the FANS UAS, thereby minimizing mucosal trauma. The Post-ureteroscopy lesion scale (PULS) score was recorded as 0.^2^ Following complete stone removal, a 6 Fr JJ stent was placed, and the procedure was completed without complications. A non-contrast CT scan obtained 24 hours postoperatively confirmed a stone-free status. The JJ stent was removed one week later.

**Conclusion:**

The use of FANS UAS in ureteral stone management proved to be safe and effective, facilitating efficient stone clearance without mucosal injury. This novel technique offers a promising adjunct to standard ureteroscopy, particularly in cases requiring precise fragmentation control and low complication risk.

## Data Availability Statement:

The data that support the findings of this study are available on request from the corresponding author.

**Artificial Intelligence Usage Statement:** The authors declared that no Artificial Intelligence Tool was used in the preparation of the manuscript.**Ethics Committee Approval:** Ethical approval was not required for this study as it is a single case report. Written informed consent was obtained from the patient for publication of this case report and any accompanying images.**Informed Consent:** Verbal and written informed consent was obtained from the patient who agreed to take part in the study.**Peer-review:** Externally peer-reviewed.**Author Contributions:** Concept – R.B.E., T.T.; Design – R.B.E., M.F.Ö.; Supervision – T.T., V.G.; Resources – V.G., S.K.K.Y., O.T.; Materials – R.B.E., M.F.Ö.; Data Collection and/or Processing – R.B.E., M.F.Ö.; Analysis and/or Interpretation – R.B.E., V.G.; Literature Search – R.B.E., M.F.Ö.; Writing – R.B.E.; Critical Review – T.T., V.G., S.K.K.Y., O.T.**Declaration of Interests:** The authors have no conflicts of interest to declare.**Video 1:** https://www.youtube.com/watch?v=-L8rG_MVFP4
